# Usability of Rapid Cholera Detection Device (OmniVis) for Water Quality Workers in Bangladesh: Iterative Convergent Mixed Methods Study

**DOI:** 10.2196/22973

**Published:** 2021-05-12

**Authors:** Theresa L Rager, Cristian Koepfli, Wasif A Khan, Sabeena Ahmed, Zahid Hayat Mahmud, Katherine N Clayton

**Affiliations:** 1 Eck Institute for Global Health University of Notre Dame Notre Dame, IN United States; 2 Infectious Diseases Division International Centre for Diarrhoeal Disease Research, Bangladesh Dhaka Bangladesh; 3 Laboratory Sciences and Services Division International Centre for Diarrhoeal Disease Research, Bangladesh Dhaka Bangladesh; 4 OmniVis Inc South San Francisco, CA United States

**Keywords:** cholera, environmental surveillance, mHealth, usability

## Abstract

**Background:**

Cholera poses a significant global health burden. In Bangladesh, cholera is endemic and causes more than 100,000 cases each year. Established environmental reservoirs leave millions at risk of infection through the consumption of contaminated water. The Global Task Force for Cholera Control has called for increased environmental surveillance to detect contaminated water sources prior to human infection in an effort to reduce cases and deaths. The OmniVis rapid cholera detection device uses loop-mediated isothermal amplification and particle diffusometry detection methods integrated into a handheld hardware device that attaches to an iPhone 6 to identify and map contaminated water sources.

**Objective:**

The aim of this study was to evaluate the usability of the OmniVis device with targeted end users to advance the iterative prototyping process and ultimately design a device that easily integrates into users’ workflow.

**Methods:**

Water quality workers were trained to use the device and subsequently completed an independent device trial and usability questionnaire. Pretraining and posttraining knowledge assessments were administered to ensure training quality did not confound trial and questionnaire

**Results:**

Device trials identified common user errors and device malfunctions including incorrect test kit insertion and device powering issues. We did not observe meaningful differences in user errors or device malfunctions accumulated per participant across demographic groups. Over 25 trials, the mean time to complete a test was 47 minutes, a significant reduction compared with laboratory protocols, which take approximately 3 days. Overall, participants found the device easy to use and expressed confidence and comfort in using the device independently.

**Conclusions:**

These results are used to advance the iterative prototyping process of the OmniVis rapid cholera detection device so it can achieve user uptake, workflow integration, and scale to ultimately impact cholera control and elimination strategies. We hope this methodology will promote robust usability evaluations of rapid pathogen detection technologies in device development.

## Introduction

Cholera is a waterborne disease caused by the bacterium *Vibrio cholerae* that has led to seven major pandemics since 1817 [1]. There are currently 47 countries affected by cholera, leading to 2.9 million cases and 95,000 deaths each year [2]. Cholera is fatal in approximately 60% of untreated cases, but the fatality rate can be reduced to a less than 1% with aggressive rehydration and electrolyte replacement [3]. In total, the disease costs an estimated US $2 billion in lost productivity and health care costs annually [2]. Cholera is a disease of inequity in that it affects the poorest and most vulnerable populations within each affected country [2]. Women of childbearing age and children aged 2 to 9 years are most at risk of infection, with about half of all cases occurring in children younger than 5 years [4,5]. These inequities are attributed to access to safe water and sanitation.

Globally, 844 million people lack access to safe drinking water, and 2.4 billion are without basic sanitation facilities, leading to 2 billion people drinking water with fecal contaminants; these inequities leave billions of people vulnerable to cholera each year [2]. Community members can be asymptomatic carriers of cholera, unknowingly excreting the bacteria and potentially contaminating water supplies [6]. The persistence of *V cholerae* in community water sources leaves local populations continuously vulnerable to infection [7]. Thus, environmental water sources must be monitored for the establishment of reservoirs.

The ability of *V cholerae* to survive in viable but nonculturable states [8] poses a potential danger to public health efforts as the highly selective media used in conventional microbiology can fail to detect the organism in environmental samples [9]. Early pathogen detection is a key component to preventing infection. International efforts call for situational analysis to identify cholera hotspots, primarily through early warning surveillance and stronger laboratory capacities to reduce cholera transmission [10]. While early warning surveillance can encompass both epidemiological and environmental surveillance techniques, using the latter to monitor community water supplies and piped water infrastructure would likely detect pathogens prior to infection and subsequently prevent outbreaks. If *V cholerae* is detected, governments and aid agencies can immediately deploy rapid response teams to distribute household water treatment products and provide community education. Additionally, environmental surveillance can inform government policymaking and implementation strategies by identifying cholera hotspots [2,11]. Rapid detection and environmental surveillance of *V cholerae* could contribute to these goals.

In recent years, the use of mobile technology has dramatically increased globally. Mobile health, or mHealth, is defined by the World Health Organization as “medical and public health practice supported by mobile devices, such as mobile phones, patient monitoring devices, personal digital assistants (PDAs), and other wireless devices” [12]. With more than 106 mobile cellular subscriptions per 100 people worldwide, mobile technologies have surpassed 100% penetrance [13]. Mobile technology has infiltrated communities around the world, uniquely positioning mHealth to address global health concerns at a low cost. Rapid pathogen detection technologies could have a significant impact on environmental surveillance efforts [7]. Current mHealth rapid pathogen detection technologies primarily use lab-on-a-chip or microfluidic technology [14]. Because of their camera, GIS, and data storage modalities, mHealth technologies are ideally suited to detect pathogens in environmental water sources and serve as a low-cost alternative to laboratory testing in low-resource settings [14-17].

**Figure 1:**
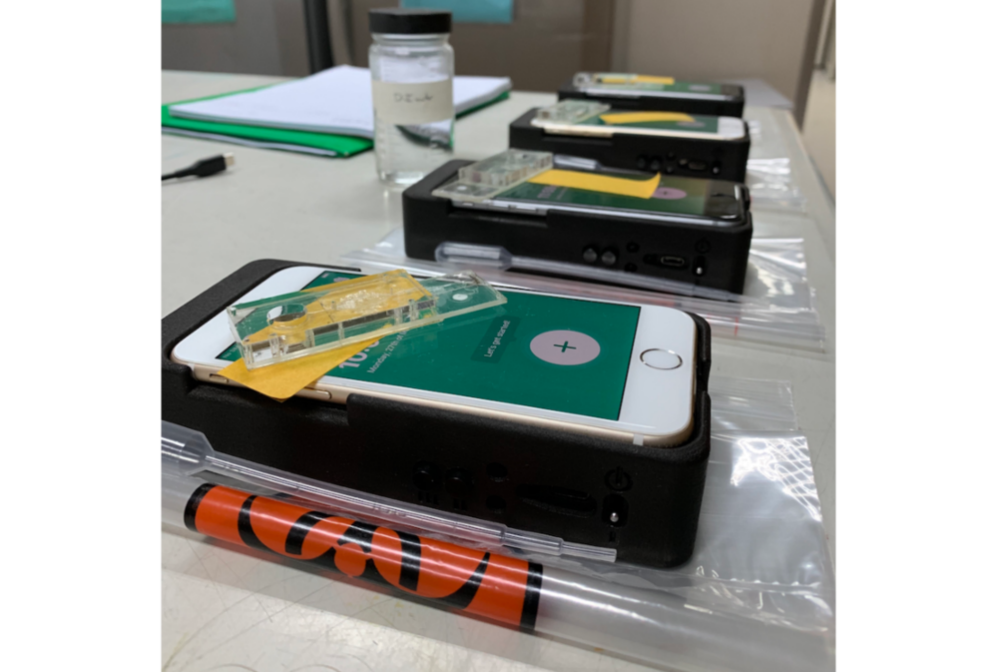
OmniVis rapid cholera detection device with inserted iPhone 6 and workstation setup for laboratory personnel trials.

Recognizing the need for increased point-of-use laboratory technologies in the field and in low-resource settings, OmniVis Inc developed a smartphone-based rapid cholera detection device (Figure 1) [18,19].

**Figure 2:**
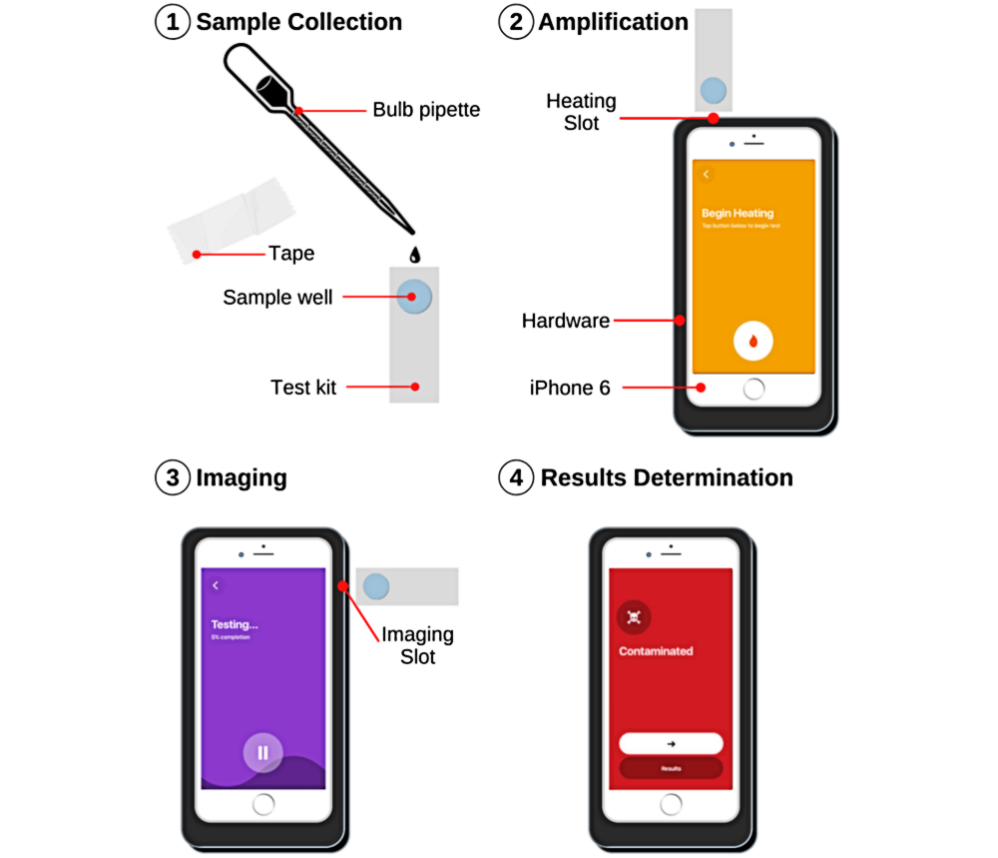
OmniVis device and app workflow: (1) environmental water sample is collected into well of single-use disposable test kit and channel is sealed, (2) test kit is inserted into hardware heating unit for loop-mediated isothermal amplification, (3) after 40 minutes, sample is placed in microscope imaging slot, (4) OmniVis app displays results: contaminated/not contaminated.

An environmental water sample (approximately 150 µL) is collected in a single-use disposable test kit and inserted into a 3D-printed hardware device with an embedded heating unit and microscope that is built to fit an iPhone 6 preloaded with the OmniVis app (Figure 2). Using particle diffusometry and loop-mediated isothermal amplification, the device detects the presence of cholera from the small water sample [18-20]. Prior to this research, the device was tested only in laboratory settings, demonstrating an estimated time-of-use of 45 minutes.

The purpose of this study was to progress the iterative prototyping process of the OmniVis rapid cholera detection device by evaluating the device’s usability with targeted end users, namely water quality workers. As defined by the International Organization for Standardization, usability is the “extent to which a product can be used by specified users to achieve specified goals with effectiveness, efficiency, and satisfaction in a specified context of use” [21]. Therefore, this study seeks to determine the effectiveness with which water quality workers can use the OmniVis device, its efficiency in detecting the presence of toxigenic cholera (*V cholerae* with the ctxA gene-encoding cholera toxin) in environmental water samples, and workers’ satisfaction in using the device. While the field of mHealth and rapid diagnostic testing for infectious disease is fast-growing, few rapid pathogen detection technologies have been developed for environmental surveillance [12,22]. Moreover, many mHealth usability studies have evaluated interventions such as behavioral change, medication adherence, or clinical decision-making technologies, but there is a literature gap for usability studies evaluating rapid pathogen detection technology [23-26]. By providing input in the device development process, end users can become cocreators and feel ownership in the device design, enabling greater uptake and integration into workflows. Therefore, it is critical to conduct usability evaluations to increase device uptake and ensure the development of culturally appropriate technologies [27]. It is important to assess the usability of the OmniVis rapid cholera detection device with targeted end users in order to contribute to environmental surveillance, control, and elimination efforts.

## Methods

### Study Location

The user-centered study took place in Dhaka, Bangladesh. Dhaka has an estimated population of 14.4 million growing by 1.02% annually and a population density of 19,447 people per square kilometer [28]. Specifically, the study was conducted in partnership with the International Centre for Diarrheal Disease Research, Bangladesh (icddr,b), which has been conducting cholera testing, research, and surveillance in Dhaka since 1960 [29].

### Participants

A convenience sample of environmental microbiology laboratory personnel (n=14) and field staff (n=11) employed by icddr,b was recruited into the study during May 2019 by the director of the environmental microbiology laboratory and director of Duaripara field operations via word of mouth at staff meetings. Eligibility was based on participant age (older than 18 years) and job role (field staff or laboratory personnel). Field staff were defined as icddr,b employees responsible for collecting environmental water samples throughout Dhaka city and the surrounding areas using sterile technique and maintaining cold chain transportation from the field to icddr,b laboratories. Laboratory personnel were defined as icddr,b employees who receive environmental water samples and conduct gold standard methods including enrichment, subculturing, and DNA amplification by polymerase chain reaction of *V cholerae* and other waterborne pathogens. Exclusion criteria included minors and nonemployees of icddr,b for adequate homogeneity of job roles. As a small-scale pilot study, only 25 participants were recruited due to funding constraints for participant compensation. All participants provided informed consent before participating in the study. Device training, device trials, and administering the usability questionnaire were conducted at the Duaripara field office for field staff participants and at the environmental microbiology laboratory for laboratory personnel participants. All participants received 820 BDT (US $10) as compensation for their time. This study was approved by the University of Notre Dame institutional review board and the icddr,b research and ethics review committees.

### Device Trials

#### Device Training

Development of a training program was not a specific objective of this research. However, participants needed to have a uniform understanding of the device prior to conducting field trials to minimize confounding variables such as familiarity with technology. Thus, prior to conducting device trials, participants were trained on how to use the device and biosafety protocols for device use. In order to identify any confounding variables resulting from poorly conducted training, the training was evaluated through a 5-question pretraining and posttraining knowledge assessment that included both true/false and open-ended questions (Multimedia Appendix 1). These assessments tested participants’ comprehension of personal protective equipment (PPE) use, sample collection, sample analysis, and proper test kit disposal. Pretraining and posttraining knowledge assessments were identical in their content. Participants completed the pretraining knowledge assessment before undergoing a device training session. Following assessment completion, the study investigator demonstrated donning PPE, collecting a water sample with single-use bulb pipette, inserting and sealing the sample into a single-use test kit, powering the device, inserting the test kit into heating and imaging slots at appropriate stages, navigating the app interface, safe disposal of the test kit and sample, and doffing PPE. Spoken English to Bangla translation of this demonstration was provided by a staff member employed by icddr,b. After completion of the training, participants completed the posttraining knowledge assessment to determine if their understanding of how to use the device improved as a result of this training.

#### Field Trials

Upon entering the laboratory setting or field office setting (hereafter, field), participants were provided with one hardware device, one iPhone 6, one test kit and an accompanying piece of tape to seal the sample well, one single-use bulb pipette for sample collection, and one biohazard bag for sample disposal. Deionized water was used in place of an environmental water sample to reduce the risk of exposure and contamination. Participant time-of-use for each trial was recorded starting immediately prior to collecting the deionized water sample and ending with disposing the test kit into the biohazard bag. Participants performed one trial relying on instructions displayed in the iPhone app and their understanding of the training. The investigator observed participant interactions with the device and recorded written field notes and photographs. Field notes included specific errors observed during each trial. Specific errors were grouped by similarity and summed. Groups were then more broadly coded into user errors and device malfunctions depending on the cause of the error. Photographs were supplied to OmniVis Inc for internal use in developing future prototypes.

#### Usability Questionnaire

A usability questionnaire (separate from the pretraining and posttraining assessments) was designed to capture user-defined errors, attitudes toward the device, and potential workflow integration pathways. Specific aims included training evaluation, device usability, workflow integration, and community education. Empirically evaluated usability questionnaires including the Mobile Phone Usability Questionnaire and the Poststudy System Usability Questionnaire were analyzed for applicable content when developing the questionnaire [30,31]. Relevant questions were adapted and reworded for applicability to the OmniVis device and testing setting. Questions were carefully ordered to follow device training and use to minimize respondent confusion or recall bias. Special attention was paid to minimize participant cognitive load by ensuring that question language was clear, specific, and nonbiased; redundant questions were omitted, and display and skip logic was used. Demographic questions were added to the end of the questionnaire to capture job role, gender, language competency, education, level of experience, and years worked at icddr,b. Both the English and Bangla versions of this study’s questionnaire were input into Qualtrics Surveys Offline Application and administered via a Fire 7 (Amazon) tablet, iPhone XR (Apple Inc), or iPhone 6 (Apple Inc). Participants completed the questionnaire immediately after the field trial via self-administration.

### Analysis

Pretraining and posttraining knowledge assessment scores and time-of-use data were input into SPSS (version 26, IBM Corp) for statistical analysis. Field notes were input into Excel spreadsheets (Microsoft Corp) for coding and cleaning before being transferred to SPSS for statistical analysis. Questionnaire data were downloaded from Qualtrics as an SPSS data sheet for statistical analysis. The sample included 25 participants (14 laboratory personnel and 11 field staff). Nonparametric tests were used for analysis due to the limited sample size.

#### Device Training

Participants were given one point per correct answer on the pretraining and posttraining knowledge assessments. The change in these scores was analyzed on SPSS based on demographics of job role, gender, and language competency.

#### Field Trials

Field notes were coded by user errors and device malfunctions. Descriptive frequencies were obtained. Aggregate counts of errors and malfunctions were analyzed across participant job role, gender, and language competency using a chi-square test of independence (α=.05) on SPSS.

#### Usability Questionnaire

Descriptive frequencies for Likert response option format and dichotomous questions were obtained. Chi-square goodness-of-fit tests and Wilcoxon signed-rank tests (α=.05) were performed on SPSS to analyze the distribution of responses around a neutral point for Likert-style questions. Dichotomous questions were analyzed using a Fisher exact test (α=.05) on SPSS to determine if there was an association between demographics and user confidence and comfort with the device.

## Results

### Participants

The sample included 25 participants, including 56% (14/25) laboratory personnel and 44% (11/25) field staff, 52% (13/25) English speakers and 48% (12/25) non-English speakers, and 80% (20/25) men and 20% (5/25) women. Participant education levels ranged from some secondary school to master’s degree (Table 1). Participant years working for icddr,b ranged from 2 months to 15 years, and their years of experience in water quality testing ranged from 3 months to 10 years. Participant demographics are further summarized in Table 1.

**Table 1:** Participant demographics (n=25).

Characteristic		Value, n (%)
**Job role**		
	Field staff	11 (44)
	Laboratory personnel	14 (56)
**Gender**		
	Male	20 (80)
	Female	5 (20)
**Language**		
	English	13 (52)
	Non-English	12 (48)
**Education^a^**		
	Some secondary school	1 (4)
	Secondary school	3 (12)
	Some university	3 (12)
	University	9 (36)
	Masters	9 (36)
**Years at icddr,b^b^**		
	<1	5 (20)
	1-5	10 (40)
	5-10	6 (24)
	10+	4 (16)
**Years of experience in water quality testing^c^**		
	<1	6 (25)
	1-5	14 (58)
	5-10	3 (13)
	10+	1 (4)

^a^Questionnaire responses of some primary school, primary school, and PhD removed as no participants indicated these as the highest level of education.

^b^icddr,b: International Centre for Diarrheal Disease Research, Bangladesh.

^c^One participant declined to answer this question, hence 24 total responses.

### Device Training

Pretraining and posttraining assessments were conducted with all participants, and assessment answers were analyzed. After the pretraining and posttraining assessments were scored, it appeared that participants were confused by questions 1 and 2 (Multimedia Appendix 1) because these questions were frequently incorrect on both the pretraining and posttraining knowledge assessments. Question 1 assessed recommended PPE use for device trials. A correct answer listed goggles, gloves, and gown. Question 2 assessed steps to collecting and sealing the environmental water sample into the test kit. A correct answer described use of a bulb pipette to fill the test kit’s water channel. Participants expressed confusion to the investigator about differences between US and Bangladesh PPE recommendations and the wording of question 2. Thus, assessments were analyzed with and without these questions. To assess knowledge gain, changes in scores from the pretraining and posttraining assessment were analyzed across demographic features. Table 2 demonstrates that the median change in scores between the pretraining and posttraining assessment was positive (ie, scores were higher on the postassessment) for all groups when analyzed with and without questions 1 and 2 (5 points vs 3 points). Positive changes suggest that participant knowledge pertaining to the necessary steps for sample collection, sample analysis, and biosafety protocols for the OmniVis device use were gained through the training. Similar scores across demographics suggest that participants obtained approximately the same level of overall understanding from the training regardless of job role, gender, or language competency.

**Table 2:** Change in assessment scores by demographics.

Change in score	Job role			Gender			Language	
	Field staff	Lab personnel	Male		Female	English		Non-English
5 points, median	2	1	2		2	2		2
3 points, median	1	1	1		1	1		1

### Field Trials

#### User Errors and Device Malfunctions

Over the course of 25 field trials, 70 user errors and 59 device malfunctions were recorded in total (ie, not per individual user). User errors are defined as improper use of the device dependent on participant actions, and device malfunctions are defined as a failure of the device independent of participant actions. Of the 70 user errors, 21 were unique, and of the 59 device malfunctions, 16 were unique. Unique errors and malfunctions were coded into the categories displayed in Table 3. Unique errors were categorized according to the perception, cognition, action framework for better evaluation [32]. Perception errors occur when users fail to perceive a stimulus (eg, visual or tactile); cognition errors include user memory, rule-based, or knowledge-based failures; and action errors occur when users are unable to act on stimuli (eg, activating a control or applying correct force).

**Table 3:** Descriptive frequencies of aggregate user errors and device malfunctions.

Error class and type			Value, n (%)		Error category				
					Perception		Cognition		Action
**User error (n=70)**									
	Incorrect tape use	5 (7)				✓			
	Incorrect test kit use	12 (17)				✓			
	Incorrect app use	11 (16)		✓					
	Incorrect test kit insertion into imaging slot	8 (11)		✓					
	Failure to perform thumb press	15 (21)						✓	
	Device powering	19 (27)						✓	
**Device malfunction (n=59)**									
	Unresponsive switches	8 (14)						✓	
	Poor test kit fit	5 (9)						✓	
	Poor fitting tape	18 (31)						✓	
	Loss of device power	27 (46)						✓	
	App closure	1 (2)						✓	

Counts of user errors and device malfunctions were compared across job role, gender, and language competency to determine if there was an association between demographics and user errors and device malfunctions. There was a significant association between language competency and error class (χ^2^_1_=4.2, *P*=.04), suggesting the number of user errors and device malfunctions differed between English and non-English speakers. Comparing aggregate counts across all 25 participants, non-English speakers experienced fewer user errors (non-English=30; English=40) but more device malfunctions than English speakers (non-English=36; English=23).

**Figure 3:**
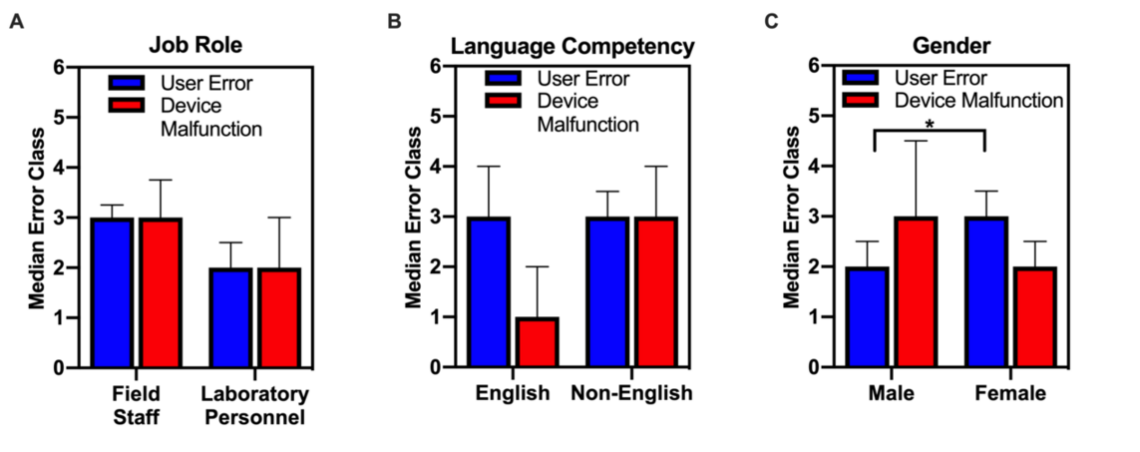
User errors and device malfunctions compared across demographics. Statistically significant (*P=.04) difference in median user errors between men and women. Errors bars represent semiinterquartile ranges.

Further analysis was performed to determine if there was a significant difference in the median number of user errors and device malfunctions per participant according to job role, gender, and language competency (Figure 3). Nonparametric tests were used for analysis because of the limited sample size. To compare the median number of errors across demographics, Mann-Whitney *U* tests (α=.05) were performed. No statistical significance was found for median number of device malfunctions or median number of user errors between field staff and laboratory personnel (Figure 3A) or English and non-English speakers (Figure 3B). Further, no statistical significance was found for the median number of device malfunctions between men and women (Figure 3C). However, there was a statistically significant difference between the median number of user errors per participant between men and women (*U*=20.5, *P*=.04). Although this difference was statistically significant, the median user errors for men and women differed by only one error per user. Furthermore, the Cramer *V* statistic suggests that the strength of this association is relatively weak (V=0.099). Therefore, this statistically significant yet relatively weak association may be confounded by unequal gender representation in the sample. Because the sample sizes were not equal (men=20, women=5), the median number of user errors by gender and the strength of this association should be evaluated in future studies with larger sample sizes and more equal gender representation.

#### Time of Use

Time of use was recorded as the time required for participants to complete one trial of a mock cholera test on the OmniVis device, beginning with picking up the test kit for sample collection and ending with disposing of the test kit in the biohazard bag. Mean time of use was 46:43 minutes (min=41:58; max=53:46). This observed time of use is less than 2 minutes greater than the hypothesized time of use (45 minutes). Compared with current gold standard laboratory methodologies that require nearly 3 days to detect cholera in environmental water samples, this mean time of use demonstrates a substantial reduction in detection time [33].

### Usability Questionnaire

#### Training Evaluation and Device Usability

Ease-of-use questions were formatted as Likert response options. English translation of these questions can be found in Multimedia Appendix 2. Question responses were coded as follows: 1=very difficult/dissimilar, 2=difficult/dissimilar, 3=neither easy/similar nor difficult/dissimilar, 4=easy/similar, 5=very easy/similar. Descriptive frequencies of these questions are shown in Table 4.

The mean and median for each question suggests that participants responded positively to the training components and device features evaluated because these descriptive frequencies are all greater than 3 (neither easy/similar nor difficult/dissimilar). Chi-square goodness of fit tests (α=.05) were performed to determine if question responses significantly differed from a uniform distribution (20% response for each answer choice). Results suggest that responses did not follow a uniform distribution and participants selected certain answer choices more than others (Table 4).

Further analysis was conducted to determine if the median response score for each question in the usability study significantly differed from 3 (neither easy/similar nor difficult/dissimilar), which represents a neutral response. A median response score greater than 3 suggests a positive response, while a response score less than 3 suggests a negative response. Wilcoxon signed-rank tests (α=.05) were performed for each Likert-style question, and results are shown in Table 4. At the 5% level of significance, the median score for each question is significantly greater than 3 (*P*<.001), suggesting that participants responded favorably to the training session and found the device easy to use overall.

**Table 4:** Training evaluation and device usability chi-square goodness of fit and 1-sample Wilcoxon signed-rank tests.

Question topic	Minimum	Median	Maximum	Chi-square value^a^	Chi-square significance	1-Sample Wilcoxon signed-rank significance^b^
Training session	3	4	5	19.2	0.001	0
Sample collection training	2	4	5	34.4	0	0
Sample seal training	2	4	5	20.0	0	0
Assembly training	2	4	5	22.0	0	0
Operate training	3	4	5	23.2	0	0
Assembly	2	4	5	20.0	0	0
Screen prompts	3	4	5	33.6	0	0
Words read	3	4	5	42.8	0	0
Color change	2	4	5	33.2	0	0
Interface similarity	2	4	5	25.6	0	0
Water collection	3	4	5	29.6	0	0
Water sample seal	3	4	5	25.6	0	0
Test kit insertion	2	4	5	15.6	0.004	0
Results read	4	4	5	42.4	0	0
Data transfer	3	4	5	42.8	0	0
Disassemble	2	4	5	29.2	0	0

^a^No cells (.0%) have expected frequencies less than 5. The minimum expected cell frequency is 5.0. Degrees of freedom for all chi-square goodness of fit tests are 4.0.

^b^Asymptotic significances are displayed for 1-sample Wilcoxon signed-rank tests. The significance level is .05.

#### User Confidence and Comfort

Nine dichotomous questions were designed to evaluate participant confidence and comfort using the OmniVis device. English translation of these questions can be found in Multimedia Appendix 2. Descriptive frequencies are displayed in Table 5.

**Table 5:** Descriptive frequencies of dichotomous questions.

Question topic	Value, n (%)	
	No	Yes
Effective test	0 (0)	25 (100)
Confidence	1 (4)	24 (96)
Device size for transport	0 (0)	25 (100)
Device size for use	0 (0)	25 (100)
Safety in the field	3 (12)	22 (88)
Safety in public	6 (24)	19 (76)
Durability	5 (20)	20 (80)
Proximity to water source	6 (24)	19 (76)
Functions	3 (12)	22 (88)

For each dichotomous question, the majority of participants responded yes, suggesting they are confident and comfortable in their ability to use the device. Participants responded most favorably to the device’s size and ability to effectively test the water sample, as indicated by the 100% yes responses. Participants expressed the least confidence and comfort in using the device in public and close to a water source, as 24% of participants answered no to these questions.

Dichotomous question responses were evaluated with Fisher exact tests (α=.05) to test for significant associations according to demographics. Statistical analysis for usability questions “Can you effectively test the water sample with the device?” “Would the device’s size be conducive to transporting it in the field or lab?” and “Would the device’s size be conducive to using it in the field or lab?” were excluded because 100% of participants responded yes to these questions. The only statistically significant result found was between gender and participant response to the question “Is the device durable enough to use in the field or lab?” (χ^2^_1_=6.25, *P*=.04). Of the female participants, 60% (3/5) responded that the device would not be durable enough to use in the field or lab while only 10% (2/20) of male participants gave the same response. Because the frequency difference between gender responses is minimal (3 female participants vs 2 male participants), these results may be confounded by a small sample size and unequal distribution of genders in the sample.

## Discussion

### Principal Findings

This study evaluates the usability of the OmniVis rapid cholera detection device with targeted end users in Dhaka, Bangladesh. Despite the importance of evaluating usability, many mHealth technologies fail to undergo such evaluations and subsequently fail to scale [12,27]. While many rapid pathogen detection technologies have undergone validity analyses, there is a literature gap of usability analyses for such technologies [14-18]. Many usability studies instead evaluate technologies that aid interventions such as behavioral change, treatment adherence, or clinical decision making [23-26]. Thus, this study can serve as a model of iterative convergent mixed methods research design for device development to further promote usability analysis of rapid pathogen detection technologies and produce rapid detection devices that better fit into user workflows for increased uptake. These results contribute to the iterative prototyping process of the OmniVis device, helping the device to achieve scale and contribute to cholera environmental surveillance. The device trials measured the effectiveness and efficiency of the device by defining common user errors and device malfunctions while also evaluating the length of time required to complete one test. The usability questionnaire elucidated water quality workers’ satisfaction with the device with questions regarding ease of use about specific components of device training and use. The questionnaire also evaluated user confidence and comfort in using the device in various settings.

Although device training was not a specific objective of this study, it was necessary to provide uniform training to participants to ensure equal knowledge of the device prior to conducting trials, and thus, minimize confounding variables. Pretraining and posttraining knowledge assessments were administered to detect if poor understanding of training confounded device trial and usability questionnaire results. Overall, participant knowledge increased as a result of the training, as demonstrated by the positive median change in scores across demographics. The only poorly understood competency was proper PPE use. Participants frequently answered question 1 (“What 3 pieces of PPE must you put on before collecting a water sample?”) incorrectly by listing different pieces of PPE other than those required by the knowledge assessment. Thus, this error suggests participants are accustomed to different PPE rather than a lack of understanding of the purpose of PPE. Nevertheless, confusion detected in assessment questions 1 and 2 indicate that the training component of this study must be reformed in order to better instruct users how to operate the device for accurate widespread use.

The objective of the device trials was to identify common user errors and device malfunctions in order to correct these errors and malfunctions in subsequent iterations of the device. Results from these device trials suggest that common user errors center around incorrect test kit use when moving from the heating to imaging phases and device powering (Figure 2, Table 3). Common device malfunctions included loss of device power and poorly fitting tape (Table 3). There was a significant association between language competency and error class in which non-English speakers experienced fewer user errors and more device malfunctions than English speakers. Because device malfunctions are defined as failures of the device independent of the participant’s actions, this association likely has a temporal confounding variable. Field staff represented a large proportion of non-English speakers in this study. Since all field staff performed their trials on the same day (trials numbers 9 to 19), device malfunctions likely resulted from previous use. For example, the 8 previous trials (trials numbers 1 to 8) likely contributed to decreased battery life, which resulted in more device malfunctions in subsequent trials and contributed to the association between non-English speakers and device malfunctions. These errors elucidate tangible improvements for future device iterations, including removal of the test kit insertion stage and redesign of device powering to make the device effective and usable in field settings.

Additionally, user errors and device malfunctions were analyzed quantitatively to determine if any particular demographic experienced errors at a higher frequency than others. By considering ease of use across demographics, device development will promote equity and accessibility to all, which is critical for widespread use in low-resource settings. The difference in median user errors for men and women was statistically significant but found to have a relatively weak association (Figure 3). Although the proportion of women in the sample was quite low, such analysis provided a gender lens to ensure that the device is not designed more favorably for men or women but rather equally usable by all genders. While there were only 5 women participants in this research, our initial studies warrant further investigation to compare error frequency between men and women in future studies. There were no statistically significant differences in median user errors or device malfunctions per participant when comparing field staff and laboratory personnel or English and non-English speakers. These results suggest that device usability is not biased according to job role or language competency.

Device trials were also used to evaluate the claim that the OmniVis device can detect toxigenic *V cholerae* in an environmental water sample in 45 minutes or less. Time-of-use data suggests that the true median test time is likely greater than 45 minutes, at an average of 46 minutes and 43 seconds. However, no participant required more than 1 hour to complete the test, suggesting that the device dramatically reduces the time required to detect toxigenic *V cholerae* in an environmental water sample. By reducing the time required to determine the presence or absence of cholera in water sources, the OmniVis device can contribute to rapid detection, early warning, and environmental surveillance of cholera in water sources—methods defined as pivotal for cholera control and elimination [10].

Usability questionnaire results clearly demonstrate that participants perceived the device use positively. Questions regarding training ease of understanding and device ease of use had median scores greater than 3, suggesting participants had positive responses (Table 4). Moreover, participants frequently responded yes to dichotomous questions, suggesting participants expressed confidence and comfort in using the device independently (Table 5). Participants responded most favorably to the device’s size and its conduciveness to transport and use in the field or lab. However, safety and durability concerns were noted. Women expressed greater concern for the durability of the device in the field (*P*=.04). Although the difference was not significant, field staff expressed increased concern for using the device in public (*P*=.06). Because field staff collect environmental water samples in public more than laboratory personnel do, they could have increased concern about publicly using an expensive device, namely an iPhone, in low-resource areas or near water sources. These concerns prompt reevaluating the use of an iPhone as opposed to a lower cost mobile platform in future iterations because safety concerns could be a barrier to uptake in this population of targeted end users.

### Limitations

Limitations of this study include sample size and testing conditions. The study involved only 25 participants, all of whom were employed by icddr,b. All reported a relatively high level of education and training. Participants represented targeted end users in Bangladesh but not all cholera-endemic countries, making the generalizability of the findings for other countries limited, but the intent of the study was to apply the findings to the OmniVis device rather than generalizing results to the population of water quality workers. In this sense, the findings provide insight into the usability of the device. In addition, only 5 women were included in the sample size. Efforts were made to include as many women as possible to achieve equal gender representation, but the sample still comprised an unequal gender distribution. The small sample size also limited the ability to pilot the pretraining and posttraining knowledge assessment and questionnaire (doing so would have depleted the available sample size). Although the questionnaire was based on the Poststudy System Usability Questionnaire, significant adaptations were made to apply the questionnaire to this study. Without such pilots, the reliability and validity of the knowledge assessment and questionnaire could not be evaluated. Additionally, field trials were completed with demonstration test kits that lacked the correct chemistry to complete a pathogen detection test. Because these demonstration test kits had to be reused in subsequent trials, environmental water samples were mimicked with deionized water. Thus, the conditions under which the device was evaluated do not perfectly mirror real testing conditions.

### Conclusion

World public health authorities have called for the evaluation of mHealth technologies in order to improve their ability to achieve scale [21]. This usability study is the first step in gaining feedback from targeted end users on the effectiveness, efficiency, and satisfaction of the OmniVis device. By employing an iterative convergent mixed methods design, this study collects user insights into device development and incorporates targeted end user attitudes and perceptions into the development of the OmniVis device [34]. Such methodology increases the likelihood of user uptake of the rapid pathogen detection technology, further contributing to environmental surveillance efforts [27]. The study demonstrated that a portable cholera detection test can be completed in less than 1 hour (mean time of use=46:43 minutes), which is a significant time reduction from the current laboratory protocol [33]. Participants responded positively to the device training and found the device easy to use at each phase of the testing process. Moreover, participants expressed confidence and comfort in using the device independently. While these results are promising for the this prototype, concerns over durability and safety require addressing in subsequent prototypes, particularly across various demographics. Future device iterations can include removing the test kit insertion stage, redesigning device powering, and adapting to a less-expensive mobile platform. Ultimately, this study will help OmniVis further develop the rapid cholera detection device so the device can better contribute to environmental surveillance efforts for cholera control and elimination. Newer generations of the OmniVis device will undergo larger user-centered trials in additional low-resource settings such as field settings in Kenya and Haiti. These studies will provide more feedback as the device is tested in different cultural and language profiles. In addition to advancing OmniVis device development, this study also promotes a feasible methodology for evaluating the usability of rapid pathogen detection technologies. Usability evaluations will help more mHealth and point-of-use environmental surveillance devices achieve scale, integration, and interoperability to further efforts to control, eliminate, and eradicate communicable diseases.

## Appendix

Multimedia Appendix 1 Pretraining and posttraining knowledge assessment (English and Bangla).

Multimedia Appendix 2 Usability questionnaire (English with coding).

Multimedia Appendix 3 Usability Questionnaire (Bangla with Coding).

## Abbreviations

icddr,b: International Centre for Diarrheal Disease Research, Bangladesh
mHealth: mobile health
PPE: personal protective equipment
