# Supplementary material for: Usability of Rapid Cholera Detection Device (OmniVis) for Water Quality Workers in Bangladesh: Iterative Convergent Mixed Methods Study
Source: J Med Internet Res. 2021 May 12;23(5):e22973. doi: 10.2196/22973 (PMC8156127; doi:10.2196/22973)
Supplement: Multimedia Appendix 1 [file jmir_v23i5e22973_app1.docx]

**Multimedia Appendix 1. Pretraining and posttraining knowledge assessment (English and Bangla).**

**Pre- and Post-Training Knowledge Assessment (English and Bangla)**

1. What three pieces of PPE must you put on before collecting a water sample?

পানির নমুনা সংগ্রহের পূর্বে ব্যক্তিগত সুরক্ষা সরঞ্জাম এর কোন ৩ টি সরঞ্জাম অবশই পরিধান করতে হবে ?

(১) ­­­­­­­­­­­­­­­­­­­____________________________________

(২) ____________________________________

(৩) ­­­­­­­­­­­­­­­­­­­____________________________________

1. What must you do before sealing the channel on the water microchip?

পানির মাইক্রোচিপ চ্যানেল বন্ধ করার পূর্বে অবশই কোন কাজটি করতে হবে?

­­­­­­­­­­­­­­­­­____________________________________________________________________________

1. True/False: You insert the chip over the heating block before the visualization block. Circle the correct answer.

সত্য/মিথ্যাঃ দৃশ্যমান ব্লকের আগে চিপটি হিটিং ব্লকের উপর সংযোগ করাতে হবে। সঠিকউত্তর গোল করুন

True সত্য

False মিথ্যা

1. True/False: The heating and light switches automatically turn on when you open the app. Circle the correct answer.

সত্য/মিথ্যাঃ আপনি যখন অ্যাপ্‌স্‌ খুলবেন, তখন স্বয়ংক্রিয়ভাবে তাপ ও আলোর সুইচ কাজ করবে । সঠিক উত্তর গোল করুন

True সত্য

False মিথ্যা

1. True/False: You can throw the microchip into a regular trash can when disassembling the device. Circle the correct answer.

ত্য/মিথ্যাঃ ডিভাইসটি খোলার পর একটি নিয়মিত ময়লার বাক্সে মাইক্রোচিপটি ফেলতে পারেন । সঠিক উত্তর গোল করুন

True সত্য

False মিথ্যা
