# Supplementary material for: Usability of Rapid Cholera Detection Device (OmniVis) for Water Quality Workers in Bangladesh: Iterative Convergent Mixed Methods Study
Source: J Med Internet Res. 2021 May 12;23(5):e22973. doi: 10.2196/22973 (PMC8156127; doi:10.2196/22973)
Supplement: Multimedia Appendix 2 [file jmir_v23i5e22973_app2.docx]

**Multimedia Appendix 2. Usability questionnaire (English with coding).**

**Usability Questionnaire (English with Coding)**

I acknowledge that I:

[ ] have read and understand the informed consent (1)

[ ] am 18 years of age or older (2)

**Specific Aim 1: Training Evaluation**

How easy or difficult was it to understand the device training session?

[ ] Very difficult (1)

[ ] Difficult (2)

[ ] Neither easy nor difficult (3)

[ ] Easy (4)

[ ] Very easy (5)

How easy or difficult was it to learn to collect a water sample into the chip?

[ ] Very difficult (1)

[ ] Difficult (2)

[ ] Neither easy nor difficult (3)

[ ] Easy (4)

[ ] Very easy (5)

How easy or difficult was it to learn to seal the water sample into the chip?

[ ] Very difficult (1)

[ ] Difficult (2)

[ ] Neither easy nor difficult (3)

[ ] Easy (4)

[ ] Very easy (5)

How easy or difficult was it to learn to assemble the device?

[ ] Very difficult (1)

[ ] Difficult (2)

[ ] Neither easy nor difficult (3)

[ ] Easy (4)

[ ] Very easy (5)

How easy of difficult was it to learn to operate the device?

[ ] Very difficult (1)

[ ] Difficult (2)

[ ] Neither easy nor difficult (3)

[ ] Easy (4)

[ ] Very easy (5)

**Specific Aim 2: Device Use Evaluation**

How easy or difficult was it to assemble the device?

[ ] Very difficult (1)

[ ] Difficult (2)

[ ] Neither easy nor difficult (3)

[ ] Easy (4)

[ ] Very easy (5)

How easy or difficult was it to understand the prompts on the device screen?

[ ] Very difficult (1)

[ ] Difficult (2)

[ ] Neither easy nor difficult (3)

[ ] Easy (4)

[ ] Very easy (5)

How easy or difficult was it to read the words on the screen?

[ ] Very difficult (1)

[ ] Difficult (2)

[ ] Neither easy nor difficult (3)

[ ] Easy (4)

[ ] Very easy (5)

How easy or difficult was it to see the colors change on the screen?

[ ] Very difficult (1)

[ ] Difficult (2)

[ ] Neither easy nor difficult (3)

[ ] Easy (4)

[ ] Very easy (5)

Is the interface similar to other mobile devices or tablets you have used?

[ ] Very dissimilar (1)

[ ] Dissimilar (2)

[ ] Neither similar nor dissimilar (3)

[ ] Similar (4)

[ ] Very similar (5)

How easy or difficult was it to collect water into the chip?

[ ] Very difficult (1)

[ ] Difficult (2)

[ ] Neither easy nor difficult (3)

[ ] Easy (4)

[ ] Very easy (5)

How easy or difficult was it to seal the water sample into the chip?

[ ] Very difficult (1)

[ ] Difficult (2)

[ ] Neither easy nor difficult (3)

[ ] Easy (4)

[ ] Very easy (5)

How easy or difficult was it to insert the chip into the device?

[ ] Very difficult (1)

[ ] Difficult (2)

[ ] Neither easy nor difficult (3)

[ ] Easy (4)

[ ] Very easy (5)

How easy or difficult was it to read the results?

[ ] Very difficult (1)

[ ] Difficult (2)

[ ] Neither easy nor difficult (3)

[ ] Easy (4)

[ ] Very easy (5)

How easy or difficult was it to transfer the data to other devices?

[ ] Very difficult (1)

[ ] Difficult (2)

[ ] Neither easy nor difficult (3)

[ ] Easy (4)

[ ] Very easy (5)

How easy or difficult was it to disassemble the device?

[ ] Very difficult (1)

[ ] Difficult (2)

[ ] Neither easy nor difficult (3)

[ ] Easy (4)

[ ] Very easy (5)

**Specific Aim 3: End User Confidence and Comfort**

Can you effectively test the water sample with the device?

[ ] Yes (1)

[ ] No (0)

Do you feel confident enough to use the device on your own?

[ ] Yes (1)

[ ] No (0)

Would the device's size be conducive to transporting it in the field?

[ ] Yes (1)

[ ] No (0)

Would the device's size be conducive to using it in the field or lab?

[ ] Yes (1)

[ ] No (0)

Would you feel safe using the device in the field or lab?

[ ] Yes (1)

[ ] No (0)

Would you feel safe using the device in public?

[ ] Yes (1)

[ ] No (0)

Is the device durable enough to use in the field?

[ ] Yes (1)

[ ] No (0)

Do you feel comfortable using the device near a water source?

[ ] Yes (1)

[ ] No (0)

Does the device have all the functions you would like it to have?

[ ] Yes (1)

[ ] No (0)

*Display This Question:*

*If Does the device have all the functions you would like it to have? = No*

What functions would you like it to have?

________________________________________________________________

**Specific Aim 4: Participant Job Responsibilities**

Select your job role below.

[ ] Field Staff (1)

[ ] Laboratory Personnel (2)

Do you collect water samples in the field?

[ ] Yes (1)

[ ] No (0)

*Display this question:*

*If Do you collect water samples in the field = Yes*

Would you prefer to use this device compared to collecting water samples in the field and transporting them to the lab?

[ ] Yes (1)

[ ] No (0)

Do you test water samples in the lab?

[ ] Yes (1)

[ ] No (0)

*Display this question:*

*If Do you test water samples in the lab = Yes*

Would you prefer to use this device compared to testing for cholera using the current lab protocol?

[ ] Yes (1)

[ ] No (0)

Do you tell communities if their water sources are contaminated?

[ ] Yes (1)

[ ] No (0)

*Skip To: Safe Drinking Habits If Do you tell communities if their water sources are contaminated? = No*

How do you tell communities their water sources are contaminated? Check all that apply.
[ ] Knocking on doors (1)

[ ] Announcements at community gathering place or church (2)

[ ] Radio (3)

[ ] Phone (4)

[ ] Other (5) ________________________________________________

How do you describe water contamination to communities?

________________________________________________________________

What diseases do you discuss? Check all that apply.

[ ] Cholera (1)

[ ] E. coli (2)

[ ] Typhoid (3)

[ ] Dysentery (4)

[ ] Hepatitis A (5)

[ ] General Diarrheal Disease (6)

[ ] Other (7) ________________________________________________

Do you educate communities about safe drinking habits?

[ ] Yes (1)

[ ] No (0)

Water purification products include chlorinated solution, purification tables, and ceramic filters. Do you advertise water purification products?

[ ] Yes (1)

[ ] No (0)

*Display This Question:*

*If Water purification products include chlorinated solution, purification tables, and ceramic filter... = Yes*

Which water purification products do you advertise? Check all that apply.

[ ] Chlorinated solution (1)

[ ] Purification tablets (2)

[ ] Ceramic filters (3)

Do you sell water purification products?

[ ] Yes (1)

[ ] No (0)

*Display This Question:*

*If Do you sell water purification products? = Yes*

Which water purification products do you sell? Check all that apply.

[ ] Chlorinated solution (1)

[ ] Purification tablets (2)

[ ] Ceramic filters (3)

**Specific Aim 5: Demographic Information**

What is your sex?

[ ] Male (1)

[ ] Female (2)

What is your age?

________________________________________________________________

What languages do you speak fluently? Check all that apply.

[ ] Bangla (1)

[ ] English (2)

What is the highest level of education your completed?

[ ] Some primary school (1)

[ ] Primary school (2)

[ ] Some secondary school (3)

[ ] Secondary school (4)

[ ] Some university (5)

[ ] University (6)

[ ] Masters (7)

[ ] PhD (8)

How many years have you worked for icddr,b?

________________________________________________________________

How many years of experience do you have with water quality testing?

________________________________
