# Supplementary material for: Usability of Rapid Cholera Detection Device (OmniVis) for Water Quality Workers in Bangladesh: Iterative Convergent Mixed Methods Study
Source: J Med Internet Res. 2021 May 12;23(5):e22973. doi: 10.2196/22973 (PMC8156127; doi:10.2196/22973)
Supplement: Multimedia Appendix 3 [file jmir_v23i5e22973_app3.docx]

**Multimedia Appendix 3. Usability Questionnaire (Bangla with Coding).**

**Usability Questionnaire (Bangla with Coding)**

আমি স্বীকার করছি যে আমি:

[ ] সম্মতিপত্রটি পড়েছি এবং বুঝেছি (১)

[ ] আমার বয়স ১৮ বা তার উপর (২)

**নির্দিষ্ট লক্ষ্য ১: প্রশিক্ষণ মূল্যায়ন**

ডিভাইস প্রশিক্ষণ অধিবেশন বুঝতে কতটা সহজ বা কঠিন ছিল?

[ ] খুব কঠিন (১)

[ ] কঠিন (২)

[ ] সহজও না আবার কঠিনও না (৩)

[ ] সহজ (৪)

[ ] খুব সহজ (৫)

চিপের মধ্যে পানির নমুনা সংগ্রহ করা শিখতে কতটা সহজ বা কঠিন ছিল?

[ ] খুব কঠিন (১)

[ ] কঠিন (২)

[ ] সহজও না আবার কঠিনও না (৩)

[ ] সহজ (৪)

[ ] খুব সহজ (৫)

চিপের মধ্যে পানির নমুনা সিল/বন্ধ করা শিখতে কত টা সহজ বা কঠিন ছিল ?

[ ] খুব কঠিন (১)

[ ] কঠিন (২)

[ ] সহজও না আবার কঠিনও না (৩)

[ ] সহজ (৪)

[ ] খুব সহজ (৫)

ডিভাইস সংযোজন করা কতটা সহজ বা কঠিন ছিল?

[ ] খুব কঠিন (১)

[ ] কঠিন (২)

[ ] সহজও না আবার কঠিনও না (৩)

[ ] সহজ (৪)

[ ] খুব সহজ (৫)

ডিভাইস চালানো শিখতে কতটা সহজ বা কঠিন ছিল?

[ ] খুব কঠিন (১)

[ ] কঠিন (২)

[ ] সহজও না আবার কঠিনও না (৩)

[ ] সহজ (৪)

[ ] খুব সহজ (৫)

নির্দিষ্ট লক্ষ্য ২: ডিভাইস ব্যবহার এর মূল্যায়ন

ডিভাইস সংযোজন করা কতটা সহজ বা কঠিন ছিল ?

[ ] খুব কঠিন (১)

[ ] কঠিন (২)

[ ] সহজও না আবার কঠিনও না (৩)

[ ] সহজ (৪)

[ ] খুব সহজ (৫)

ডিভাইসের পর্দার লেখা বোঝার ক্ষেত্রে কতটা সহজ বা কঠিন ছিল ?

[ ] খুব কঠিন (১)

[ ] কঠিন (২)

[ ] সহজও না আবার কঠিনও না (৩)

[ ] সহজ (৪)

[ ] খুব সহজ (৫)

ডিভাইস এর পর্দায় শব্দ পড়া কতটা সহজ বা কঠিন ছিল ?

[ ] খুব কঠিন (১)

[ ] কঠিন (২)

[ ] সহজও না আবার কঠিনও না (৩)

[ ] সহজ (৪)

[ ] খুব সহজ (৫)

পর্দায় রঙ পরিবর্তন দেখা কতটা সহজ বা কঠিন ছিল ?

[ ] খুব কঠিন (১)

[ ] কঠিন (২)

[ ] সহজও না আবার কঠিনও না (৩)

[ ] সহজ (৪)

[ ] খুব সহজ (৫)

এটার ইন্টারফেস কী অন্যান্য ফোনের/ ট্যাবলেট এর মতো যা আপনি ব্যবহার করেছেন?

[ ] খুব বিসদৃশ (১)

[ ] বিসদৃশ (২)

[ ] সদৃশ না আবার বিসদৃশ না (৩)

[ ] সদৃশ (৪)

[ ] খুব সদৃশ (৫)

চিপের মধ্যে পানি সংগ্রহ করা কতটা সহজ বা কঠিন ছিল?

[ ] খুব কঠিন (১)

[ ] কঠিন (২)

[ ] সহজও না আবার কঠিনও না (৩)

[ ] সহজ (৪)

[ ] খুব সহজ (৫)

চিপের মধ্যে পানির নমুনা সিল/ বন্ধ করা কতটা সহজ বা কঠিন ছিল?

[ ] খুব কঠিন (১)

[ ] কঠিন (২)

[ ] সহজও না আবার কঠিনও না (৩)

[ ] সহজ (৪)

[ ] খুব সহজ (৫)

ডিভাইস এর ভিতরে চিপ ‍ঢোকানো কতটা সহজ বা কঠিন ছিল?

[ ] খুব কঠিন (১)

[ ] কঠিন (২)

[ ] সহজও না আবার কঠিনও না (৩)

[ ] সহজ (৪)

[ ] খুব সহজ (৫)

ফলাফল পড়তে কতটা সহজ বা কঠিন ছিল ?

[ ] খুব কঠিন (১)

[ ] কঠিন (২)

[ ] সহজও না আবার কঠিনও না (৩)

[ ] সহজ (৪)

[ ] খুব সহজ (৫)

অন্য ডিভাইস এ ডাটা স্থানান্তর কতটা সহজ বা কঠিন ছিল ?

[ ] খুব কঠিন (১)

[ ] কঠিন (২)

[ ] সহজও না আবার কঠিনও না (৩)

[ ] সহজ (৪)

[ ] খুব সহজ (৫)

ডিভাইস খোলা/ আলাদা করতে কতটা সহজ বা কঠিন ছিল ?

[ ] খুব কঠিন (১)

[ ] কঠিন (২)

[ ] সহজও না আবার কঠিনও না (৩)

[ ] সহজ (৪)

[ ] খুব সহজ (৫)

**নির্দিষ্ট লক্ষ্য ৩: ব্যাবহারকারিদের বিশ্বাস এবং স্বাচ্ছ্যন্দতা**

আপনি কি এই ডিভাইস দিয়ে কার্যকরভাবে পানি পরিক্ষা করতে পারেন ?

[ ] হ্যাঁ (১)

[ ] না (0)

আপনি নিজে নিজে ডিভাইসটি ব্যবহার করতে যথেষ্ট আত্মবিশ্বাসী বোধ করেন?

[ ] হ্যাঁ (১)

[ ] না (0)

ডিভাইসটির আয়তন কি মাঠ-পর্যায়ে পরিবহনের ক্ষেত্রে সুবিধাজনক?

[ ] হ্যাঁ (১)

[ ] না (0)

**ডিভাইসটির আয়তন কি মাঠ-পর্যায়ে বা পরীক্ষাগারে ব্যবহার এর ক্ষেত্রে সুবিধাজনক?**

[ ] হ্যাঁ (১)

[ ] না (0)

আপনি কি মাঠ-পর্যায়ে বা পরীক্ষাগারে এই ডিভাইস ব্যবহার করে নিরাপদ বোধ করবেন ?

[ ] হ্যাঁ (১)

[ ] না (0)

জনসমাগমে কি এই ডিভাইস ব্যবহার করে নিরাপদ বোধ করবেন ?

[ ] হ্যাঁ (১)

[ ] না (0)

এই ডিভাইসটি কি মাঠ-পর্যায়ে ব্যবহার করার জন্য যথেষ্ট টেকসই ?

[ ] হ্যাঁ (১)

[ ] না (0)

আপনি কি এই ডিভাইসটি পানির উৎসের কাছাকাছি জায়গায় ব্যবহার করতে স্বাচ্ছ্যন্দবোধ করেন ?

[ ] হ্যাঁ (১)

[ ] না (0)

আপনি যে ধরনের বৈশিষ্ট্য / কার্যক্ষমতা পছন্দ করেন তার সব কি এই ডিভাইসে রয়েছে?

[ ] হ্যাঁ (১)

[ ] না (0)

*প্রশ্নটি দেখান:*

**যদি আপনি যে ধরনেরবৈশিষ্ট্য / কার্যক্ষমতা পছন্দ করেন তার সব কি এই ডিভাইসে রয়েছে? = না**

আপনি এটিতে কি কি বৈশিষ্ট্য / কার্যক্ষমতা পছন্দ করবেন ?

____________________________________________________________

নির্দিষ্ট লক্ষ্য (৪): অংশগ্রহনকারীর দায়িত্বসমূহ

আপনার কাজের ধরন নির্বাচন করুন

[ ] মাঠ কর্মী (১)

[ ] পরীক্ষাগার কর্মী (২)

আপনি কি মাঠ পর্যায়ে পানির নমুনা সংগ্রহ করেন ?

[ ] হ্যাঁ (১)

[ ] না (0)

***প্রশ্নটি প্রদর্শন করুন আপনি যদি মাঠ পর্যায়ে পানির নমুনা সংগ্রহ করেন = হ্যাঁ***

আপনি কি এই ডিভাইসটি পানির নমুনা সংগ্রহ করে গবেষণাগারে পাঠানো অপেক্ষা মাঠ-পর্যায়ে পানির নমুনা পরীক্ষা করাকে তুলনামূলকভাবে বেশি পছন্দ করবেন?

[ ] হ্যাঁ (১)

[ ] না (0)

আপনি কি গবেষণাগারে পানি পরীক্ষা করেন ?

[ ] হ্যাঁ (১)

[ ] না (0)

প্রশ্নটি প্রদর্শন করুন আপনি যদি পরীক্ষাগারে পানির নমুনা পরীক্ষা করেন = হ্যাঁ

বর্তমানে পরীক্ষাগারে যে পদ্ধতি ব্যাবহার করে কলেরা পরীক্ষা করেন তার তুলনায় আপনি কি এই যন্ত্র দিয়ে আরো বেশি পছন্দ করবেন ?

[ ] হ্যাঁ (১)

[ ] না (0)

আপনি কি লোকালয় এর মধ্যে পানির উৎস দূষিত হওয়ার কথা বলেন?

[ ] হ্যাঁ (১)

[ ] না (0)

**যদি আপনি কি লোকালয় এর মধ্যে পানির উৎস দূষিত হওয়ার কথা বলেন? = না হয় তবে *নিরাপদ পানি পানের অভ্যাস এ যান***

আপনি লোকালয় এ পানির উৎস দূষিত হওয়ার কথা কিভাবে বলেন? (প্রযোজ্য সবগুলো চিহ্নিত করুন)

[ ] দরজায় কড়া নাড়া (১)

[ ] এ গনজমায়েতে/ধর্মালয়ে ঘোষণা (২)

[ ] রেডিও (৩)

[ ] ফোন (৪)

[ ] অন্যান্য (৫)______________________________________________

পানির দূষিত হওয়ার কথা আপনি লোকালয় এ কিভাবে বর্ণনা করেন?

__________________________________________________________

কি ধরনের রোগ নিয়ে আলাপ করেন ? প্রযোজ্য সবগুলো চিহ্নিত করুন

[ ] কলেরা (১)

[ ] ই কোলাই (এক ধরনের ব্যাকটেরিয়া) (২)

[ ] টাইফয়েড (৩)

[ ] আমাশয় (৪)

[ ] হেপাটাইটিস এ (৫)

[ ] সাধারণ ডায়রিয়া রোগ (৬)

[ ] অন্যান্য (৭) ________________________________________________

আপনি কি লোকালয় এর মানুষদের নিরাপদ পানি পানের অভ্যাস নিয়ে কোন শিক্ষা প্রদান করেন ?

[ ] হ্যাঁ (১)

[ ] না (0)

পানি বিশুদ্ধকরন পণ্যের মধ্যে ক্লোরিনের সল্যুশন, পানিবিশুদ্ধকরন বড়ি এবং সিরামিক ফিল্টার অন্তভুক্ত । আপনি কি পানি বিশুদ্ধকরন পণ্যের প্রচারনা করেন ?

[ ] হ্যাঁ (১)

[ ] না (0)

*প্রশ্নটি দেখান যদি*পানি বিশুদ্ধকরন পণ্যের মধ্যে ক্লোরিনেরসল্যুশন,পানি বিশুদ্ধকরন *বড়ি এবং সিরামিক ফিল্টারঅন্তভুক্ত.... = হ্যাঁ*

পানি বিশুদ্ধকরন কোন পণ্যের প্রচারে কাজ করেন ? প্রযোজ্য সবগুলো চিহ্নিত করুন

[ ] ক্লোরিনের সল্যুশন (১)

[ ] পানি বিশুদ্ধকরন বড়ি (২)

[ ] সিরামিক ফিল্টার (৩)

আপনি কি পানি বিশুদ্ধকরন পণ্য বিক্রয় করেন ?

[ ] হ্যাঁ (১)

[ ] না (0)

***প্রশ্নটি দেখান যদি আপনি কি পানিবিশুদ্ধকরন পণ্যগুলো বিক্রয় করেন? =হ্যাঁ***

নিচের কোন কোন পানি বিশুদ্ধকরন পণ্যগুলো আপনি বিক্রয় করেন ? প্রযোজ্য সবগুলো চিহ্নিত করুন

[ ] ক্লোরিনের সল্যুশন (১)

[ ] পানি বিশুদ্ধকরন বড়ি (২)

[ ] সিরামিক ফিল্টার(৩)

**নির্দিষ্ট লক্ষ্য (৫): জনসংখ্যা তাত্ত্বিক তথ্য**

আপনার লিঙ্গ ?

[ ] পুরুষ (১)

[ ] মহিলা (২)

আপনার বয়স কত ?

________________________________________________________________

কোন ভাষায় আপনি কথা বলতেপারদর্শী ? প্রযোজ্য সবগুলো চিহ্নিত করুন

[ ] বাংলা (১)

[ ] ইংলিশ (২)

আপনার শিক্ষাগত যোগ্যতার সর্বোচ্চ কোন স্তর শেষ করেছেন?

[ ] প্রাথমিক বিদ্যালয়ের কয়েকটি স্তর (১)

[ ] প্রাথমিক বিদ্যালয় (২)

[ ] মাধ্যমিক বিদ্যালয়ের কয়েকটি স্তর (৩)

[ ] মাধ্যমিক বিদ্যালয় (৪)

[ ] বিশ্ববিদ্যালয়ের কয়েকটি স্তর (৫)

[ ] বিশ্ববিদ্যালয় (৬)

[ ] স্নাতকোত্তর (৭)

[ ] ডক্টরেট (৮)

আপনি কত বছর যাবত আইসিডিডিআরবি,তে কাজ করছেন ?

________________________________________________________________

পানির গুনাগুন পরীক্ষা বিষয়ে আপনার কত বছরেরঅভিজ্ঞতা রয়েছে ?

___________________________________________________________
